# Protective effects of a unique combination of nutritionally active ingredients on risk factors and gene expression associated with atherosclerosis in C57BL/6J mice fed a high fat diet†

**DOI:** 10.1039/d0fo02867c

**Published:** 2021-03-09

**Authors:** Joe W. E. Moss, Jessica O. Williams, Wijdan Al-Ahmadi, Victoria O'Morain, Yee-Hung Chan, Timothy R. Hughes, Juan B. Menendez-Gonzalez, Alhomidi Almotiri, Sue F. Plummer, Neil P. Rodrigues, Daryn R. Michael, Dipak P. Ramji

**Affiliations:** Cardiff School of Biosciences, Cardiff University Sir Martin Evans Building Museum Avenue Cardiff CF10 3AX UK Ramji@Cardiff.ac.uk +0044 (0)2920874116 +0044 (0)29 20876753; Systems Immunity Research Institute, School of Medicine, Cardiff University Cardiff CF14 4XN UK; European Cancer Stem Cell Research Institute, Cardiff School of Biosciences, Cardiff University Hadyn Ellis Building Maindy Road Cardiff CF24 4HQ UK; Cultech Limited Unit 2 Christchurch Road Baglan Industrial Park Port Talbot SA12 7BZ UK

## Abstract

Atherosclerosis, an inflammatory disorder of the vasculature and the underlying cause of cardiovascular disease, is responsible for one in three global deaths. Consumption of active food ingredients such as omega-3 polyunsaturated fatty acids, flavanols and phytosterols has many beneficial effects on cardiovascular disease. However, their combined actions on the risk factors for atherosclerosis remains poorly understood. We have previously shown that a formulation containing each of these active components at physiologically relevant doses modulated several monocyte/macrophage processes associated with atherosclerosis *in vitro*, including inhibition of cytokine-induced pro-inflammatory gene expression, chemokine-driven monocyte migration, expression of M1 phenotype markers, and promotion of cholesterol efflux. The objectives of the present study were to investigate whether the protective actions of the formulation extended *in vivo* and to delineate the potential underlying mechanisms. The formulation produced several favourable changes, including higher plasma levels of HDL and reduced levels of macrophages and myeloid-derived suppressor cells in the bone marrow. The mRNA expression of *liver-X-receptor-α*, *peroxisome proliferator-activated receptor-γ* and *superoxide dismutase-1* was induced in the liver and that of *interferon-γ* and the *chemokine (C–X–C motif) ligand 1* decreased, thereby suggesting the potential mechanisms for many beneficial effects. Other changes were also observed such as increased plasma levels of triglycerides and lipid peroxidation that may reflect potential activation of brown fat. This study provides new insights into the protective actions and the potential underlying mechanisms of the formulation *in vivo*, particularly in relation to risk factors together with changes in systemic inflammation and hepatic lipid alterations associated with atherosclerosis and metabolic syndrome, and supports further assessments in human trials.

## Abstract 2

A combination of nutritionally active ingredients containing omega-3 polyunsaturated fatty acids, flavanols and phytosterols produce many beneficial effects on atherosclerosis-associated risk factors and gene expression in mice fed a high fat diet.

## Graphical abstract

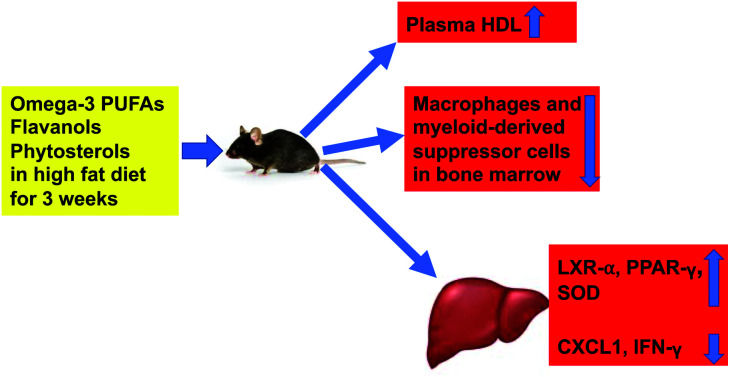

## 1. Introduction

Cardiovascular disease (CVD), including myocardial infarction (MI) and cerebrovascular accident, is the leading cause of global morbidity and mortality.^1^ According to the Heart Disease and Stroke Statistics 2020,^1^ CVD accounted for 17.8 million deaths per year in 2017. This figure is expected to increase to more than 23.6 million by 2030 in part due to a rise in risk factors such as obesity and diabetes together with the acquisition of a sedentary Westernized lifestyle in developing countries.^1–3^ This will continue to impose enormous economic burden on the healthcare system and hence it is important to develop new approaches for the prevention of CVD. The annual direct and indirect cost of CVD in the United States from 2014 to 2015 was estimated to be $351.3 billion.^1^ It is projected that between 2015 and 2035, this will remain relatively stable for 18- to 44-year olds, increase slightly for 45- to 64-year olds and increase substantially for those that are over 65 years of age.^1^

Atherosclerosis, an inflammatory disease of medium and large arteries associated with the accumulation of lipids and fibrous elements, is the underlying cause of CVD.^2–4^ The accumulation of Apolipoprotein (Apo)B-containing lipoproteins, particularly low-density lipoproteins (LDL) in the intima of arteries is a major instigator of atherosclerosis.^2–4^ The modification of LDL, particularly oxidation to form oxidized (ox)-LDL, together with other risk factors causes activation or dysfunction of the nearby endothelial cells.^2–4^ This is associated with an inflammatory response characterized by the secretion of chemokines by endothelial cells and increased expression of adhesion proteins on their cell surface.^2–4^ The net result of such changes is the recruitment of immune cells, particularly monocytes, to the site of oxLDL accumulation in the intima of arteries.^2–4^ The recruited monocytes then differentiate into macrophages, which begin to uptake oxLDL *via* receptor- and non-receptor-mediated mechanisms to transform into lipid-laden foam cells.^2–4^ Accumulation of intracellular cholesterol due to such increased uptake of oxLDL together with reduced efflux of the sterol to high density-lipoprotein (HDL) particles, and hence decreased reverse cholesterol transport to the liver, eventually leads to the lysis of the foam cells by apoptosis and necrosis.^2–4^ This results in the development of a lipid-rich necrotic core in the arterial wall which then causes a chronic inflammatory response regulated by cytokines.^2,5–8^ Smooth muscle cells migrate from the media to the intima and produce an extracellular matrix (ECM) to stabilize the atherosclerotic plaque.^2–4^ Excessive degradation of the ECM by a range of proteases produced by the different cells in the plaque under inflammatory conditions causes plaque destabilization, which can ultimately lead to its rupture and subsequent thrombosis and clinical complications such as MI.^2–6^ Approaches that modulate lipid homeostasis and the inflammatory response represent promising avenues for therapeutic intervention.

Current therapies against atherosclerosis such as statins, which lower LDL-cholesterol and have additional pleiotropic actions, are associated with considerable residual risk for CVD.^2–4^ In addition, many patients are unable to achieve target LDL-cholesterol levels even with the maximum dose of statins or are susceptible to the various side effects such as rhabdomyolysis and increased risk for developing type 2 diabetes.^2,3^ In terms of alternative therapies, some success has been seen recently with the cholesterol absorption inhibitor ezetimibe, and expensive monoclonal antibodies that target proprotein convertase subtilisin/kexin type 9, a protease involved in the degradation of the LDL receptor, and the cytokine interleukin (IL)-1β.^2,3^ However, many promising pharmaceutical agents for the treatment of CVD identified from various drug discovery programs [*e.g.* inhibitors of cholesterol ester transfer protein, lipoprotein-associated phospholipase A2] have produced disappointing outcomes in clinical trials because of off-target effects and/or safety concerns,^2,3,9^ thereby emphasizing the need to develop other agents for disease prevention and therapeutic intervention.

Consumption of various food ingredients is associated with favourable cardiovascular health.^3,4^ For example, fish oils rich in omega-3 polyunsaturated fatty acids (PUFA), such as eicosapentaenoic acid (EPA) and docosahexaenoic acid (DHA), reduce CVD *via* multiple actions, including reducing plasma cholesterol levels and dampening inflammation.^3,4^ Indeed, the recent Reduction of Cardiovascular Events With Icosapent Ethyl–Intervention Trial (REDUCE-IT) of 8179 participants showed that in patients with elevated levels of triglycerides (TG) despite the use of statins, the risk of CVD was significantly lower in those receiving 2 g of icosapent ethyl (ethyl-EPA) twice daily compared to the placebo control.^10^ Similarly, flavanols such as catechins present in cocoa have been shown to reduce risk factors for CVD in pre-clinical studies and small clinical trials.^3^ Phytosterols (PS) are plant sterols and stanols that compete with cholesterol for the intestinal uptake of dietary and biliary cholesterol.^3,4^ An epidemiological study involving 22 256 individuals showed direct correlation between diets rich in PS and reduced plasma LDL.^11^ Similarly, a *meta*-analysis of 41 trials demonstrated approximately 10% reduction of plasma LDL with daily consumption of 2 g of PS.^12^

Although many studies have investigated the cardiovascular benefits of individual food components, very few have analyzed combinations in their natural context that delivers physiological doses of the active ingredients.^3,4^ We have previously investigated the action of a combination of omega-3 PUFA-rich fish oil, flavanol-rich cocoa extract and mixed PS in a readily absorbable emulsified format^13,14^ at a physiologically relevant dose on several key monocyte/macrophage processes associated with atherosclerosis *in vitro*.^15^ The formulation produced inhibition of chemokine-driven monocyte migration, cytokine-induced expression of key genes implicated in inflammation and macrophage polarization to a pro-inflammatory M1 phenotype, and enhanced cholesterol efflux from foam cells.^15^ However, it is important to extend the findings on the beneficial effects *in vitro* to an *in vivo* context given the potential differences in metabolic transformations and other actions that are likely to exist. The effect of the formulation *in vivo* has not been analyzed and formed the focus of the current acute study in C57BL/6J mice fed a high fat diet (HFD) for 3 weeks. The experimental approach was similar to our previous study in C57BL/6J mice fed a HFD for 2 weeks that demonstrated many beneficial actions of probiotics.^16^ The impact on several risk factors associated with atherosclerosis and metabolic syndrome was investigated, including the weight of mice and various fat depots; plasma levels of lipoproteins, reactive oxygen species (ROS) and key cytokines; hematopoietic stem and progenitor cell profiles in the bone marrow; and the expression of disease-associated genes in the liver. Our hypothesis was that the formulation will produce beneficial changes in risk factors associated with atherosclerosis and metabolic syndrome such as reduced plasma levels of LDL/very low-density lipoprotein (VLDL), pro-inflammatory cytokines and markers of oxidative stress, increased plasma levels of HDL and anti-inflammatory cytokines, and anti-inflammatory immune cell profile in the bone marrow.

## 2. Materials and methods

### 2.1. Materials

All chemicals were from Sigma-Aldrich (Poole, UK) unless otherwise stated.

### 2.2. Animal experiments

All studies and protocols were approved by the Cardiff University Institutional Ethics Review Committee and the United Kingdom Home Office, and experiments were performed in accordance with the Guide for the Care and Use of Laboratory Animals (NIH publication no. 85-23, revised 1996; experimental licence 30/3365).

Male C57BL/6J mice were housed in a light and temperature-controlled facility (lights on 7 am to 7 pm, 22 °C). A total of 13 mice (8-week-old) were included in this study and assigned to two groups in standard cages and received daily gavage in the morning of either the vehicle (6 mice) or the formulation (7 mice) for 3 weeks with *ad libitum* access to a HFD [21% (w/w) pork lard and 0.15% (w/w) cholesterol; Special Diet Services, Witham, UK].^16^ The number of animals and the duration of HFD feeding are similar to our previous study on probiotics (6 mice per group and 2-week HFD feeding) that demonstrated many beneficial anti-atherogenic changes.^16^ The formulation delivered 410 mg kg^−1^ of omega-3 PUFAs EPA/DHA, 410 mg kg^−1^ of PS and 41 mg kg^−1^ of flavanols in an emulsified format^13,14^ (equating to approximate human doses of 2 g EPA/DHA, 2 g PS and 200 mg flavanols according to Nair and Jacob).^17^ The key active ingredients in the formulation were EPA, DHA, catechins, stigmasterol, campsterol, sitosterol and brassicasterol.^15^ The vehicle control was xanthan gum used as an emulsifying agent in the formulation.

Body weight was determined at the start of the study, following assignment to the two groups, and then approximately every 2 days. Food intake was controlled (*i.e.* the same weight of food was given per mouse per day), thereby allowing weight gain over time between the formulation and control mice to be determined. At the end of the study, all mice were sacrificed under anaesthesia (carbon dioxide) by cardiac puncture and death confirmed by palpitation. Blood from cardiac puncture was collected into tubes containing heparin and the plasma obtained by centrifugation (12 000*g*) for 10 min. Rear legs were removed for bone marrow extraction and placed into tubes containing PBS supplemented with 2% (v/v) heat-inactivated fetal calf serum (HI-FCS) for cell population analysis. The fat pads and organs were weighed and snap frozen in liquid nitrogen and stored at −80 °C.

### 2.3. Plasma lipid analysis

The plasma levels of total cholesterol, LDL/VLDL and HDL were determined using the Cholesterol Assay Kit -HDL and LDL/VLDL (ab65390) and TG were measured using the Triglyceride Assay Kit (ab65336) according to the manufacturer's instructions (Abcam, Cambridge, UK).

### 2.4. Plasma ROS and malondialdehyde (MDA) levels

The plasma levels of ROS and MDA were determined using the OxiSelect *in vitro* ROS/RNS Assay Kit (ROS quantification) and TBARS Assay Kit (MDA quantification) according to the manufacturer's instructions (Cell Biolabs Inc, San Diego, USA).

### 2.5. Plasma cytokine levels

Plasma cytokine levels were determined by the Central Biotechnology Services (School of Medicine, Cardiff University, UK) using a V-PLEX Plus Pro-inflammatory Panel 1 Mouse Kit that provides assay-specific components for the quantitative determination of multiple cytokines in biological samples (*e.g.* 50 μl plasma) according to the manufacturer's instructions (Meso Scale Discovery, Maryland, USA).

### 2.6. Analysis of hematopoietic cell populations within the bone marrow

The tibia and femur were homogenized with PBS supplemented with 2% (v/v) HI-FCS and filtered through a 70 μm filter. The total number of cells was then determined and 10 million, 8 million and a further 1 million cells were used to analyze the signaling lymphocytic activation molecule (SLAM), progenitor cell populations and cell lineage populations respectively. These numbers reflect the abundance of different populations in the bone marrow and allow accurate determination of their frequency by counting all possible events.

The cells were stained at 4 °C for 30 min with a biotinylated mix of lineage marker antibodies (CD3e, CD4, CD8a, B220, Gr1, Mac1, Ter119) present within the SLAM and progenitor cell populations (see Table S1† for sources of all the antibodies) along with specific antibodies. The antibodies for the analysis of the SLAM cell population were phycoerythrin (PE)/cyanine7 (Cy7)-conjugated anti-mouse CD150; FITC-conjugated anti-mouse CD48; allophycocyanin (APC)-conjugated anti-mouse c-Kit; and PE-conjugated anti-mouse stem cell antigen (Sca)-1. The progenitor cell population was analysed using PE-conjugated anti-mouse CD127; PE/Cy7-conjugated anti-mouse CD16/32; FITC-conjugated anti-mouse CD34; APC-conjugated anti-mouse c-Kit; and APC/Cy7-conjugated anti-mouse Sca-1. After initial staining, the cells were washed with 2% (v/v) PBS and resuspended in PerCP-Cy5.5-conjugated streptavidin (eBioscience, Altrincham, UK) and incubated for a further 15 min at 4 °C. For lineage cell populations, the cells were incubated at 4 °C for 20 min with APC-conjugated anti-mouse B220, FITC-conjugated anti-mouse CD3, PE/Cy7-conjugated anti-mouse Gr1, PE-conjugated anti-mouse Mac1 and APC/Cy7-conjugated anti-mouse Ter119. Following incubation, the cells were washed and resuspended in 2% (v/v) PBS.

The samples were vortexed, filtered (pore size 40 μm) and DAPI stain (20 μg ml^−1^) added at a 1 : 100 dilution to all of the SLAM, progenitor and lineage samples in order to identify viable cells. A BD LSR Fortessa 4 lasers flow cytometer was then used to assess the composition of the cell populations and all possible frequencies were collected from each sample. The markers of the hematopoietic cell populations analyzed in this study were Lin^−^ Sca-1^+^ c-Kit^+^ (LSK), CD150^+^ CD48^−^ LSK (hematopoietic stem cell; HSC), CD150^−^ CD48^−^ LSK (multipotent progenitors; MPP), CD150^−^ CD48^+^ LSK (hematopoietic progenitor cell I; HPC-I) and CD150^+^ CD48^+^ LSK (hematopoietic progenitor cell II; HPC-II) in the SLAM class; Lin^−^ Sca-1^−^ c-Kit^+^ (LK), CD34^+^ CD16/32^−^ LK (common myeloid progenitor; CMP), CD34^−^ CD16/32^−^ LK (megakaryocyte-erythroid progenitor; MEP), CD34^+^ CD16/32^+^ LK (granulocyte-macrophage progenitor; GMP) and CD127^+^ (common lymphoid progenitor; CLP) in the progenitor class; and GR1^+^ Mac1^−^ (granulocytes), GR1^+^ Mac1^+^ (myeloid-derived suppressor cells; MDSC), GR1^−^Mac1^+^ (macrophages), B220^+^ (B-cells), CD3^+^ (T-cells) and Ter119 + (red blood cells) in the lineage class.

### 2.7. Liver gene expression

Approximately 50 mg of liver was homogenized with 1 ml of RiboZol™ (Amresco LLC, Ohio, USA) and total RNA isolated according to the manufacturer's instructions (Amresco LLC, Ohio, USA). The quality of RNA was routinely assessed by determining the A260 : A280 and A260 : A230 ratios using NanoDrop 2000 and size-fractionation of an aliquot by agarose gel electrophoresis. The RNA (1000 ng) was then reverse transcribed using Moloney Murine Leukemia Virus reverse transcriptase according to the manufacturer's instructions (Promega, Southampton, UK). The cDNA (10 ng) was then used for quantitative polymerase chain reaction (qPCR) using Atherosclerosis RT^2^ Profiler PCR Arrays (Qiagen, Manchester, UK), which contains primers for 84 atherosclerosis-associated and five housekeeping genes together with three negative, three positive and one genomic DNA contamination controls, with SYBR Green JumpStart™ Taq Readymix™ for qPCR. The qPCR array involved a two-step amplification (melting at 95 °C for 15 s and simultaneous annealing/extension at 60 °C for 60 s) for 45 cycles in the LightCycler® 96 Real-Time PCR System (Roche, Welwyn Garden City, UK). Gene transcript levels were calculated using the comparative Ct method and normalized to five housekeeping genes [*β-actin*; β-2-microglobulin (*B2m*); *Gapdh*; β-glucuronidase (*Gusb*); and heat shock protein HSP90αβ1 (*Hsp90ab1*)].

### 2.8. *In vitro* experiments

Culturing of the human acute monocytic leukemia THP-1 cell line, its differentiation into macrophages and treatment with vehicle or physiological concentration of the formulation or various active ingredients were carried out as previously described.^15^ Production of ROS *in vitro* was determined using a 2′7′-dichlorofluorescin diacetate (DCFDA) Cellular ROS Detection Assay Kit (ab113851) according to the manufacturer's instructions (Abcam, Cambridge, UK). *tert*-Butyl hydroperoxide (TBHP) was used as a positive control for ROS production in cells.

### 2.9. Data analysis

The normality of the data sets was tested using Shapiro–Wilk test, histograms and Q–Q plots and any data transformations have been stated. Data values outside two standard deviations of the mean were classed as outliers and removed before statistical analysis (the *N* numbers are indicated in the appropriate tables or figure legends). To compare the means of two groups, either a *t*-test (normal data distribution) or a Mann–Whitney test (non-normal distribution) was used. To compare the means of multiple groups, a one-way analysis of variance (ANOVA) was used with appropriate *post-hoc* tests (Tukey's or Dunnett if there was equal variation between the different test groups or Dunnett T3 or Games-Howell *post-hoc* in cases of unequal variation). A generalised linear model (GLM) was used to assess a combination of continuous and categorical variables simultaneously and to avoid pseudoreplication. Significance was defined as *p* ≤ 0.05.

## 3. Results

### 3.1. The effect of the formulation on weight parameters and plasma lipid, cytokine and oxidant profiles: beneficial effects on plasma cholesterol with increased levels of HDL

Mouse weight gain positively correlated with time (Fig. S1†) with the mice gaining approximately 0.09 g per day resulting in a total average increase in weight of 1.89 g (*p* < 0.001; adj-*R*^2^ = 0.86) over the duration of the study. There was no significant difference in the rate of weight gain or the final weight in mice receiving a HFD and the formulation compared to those on HFD and the vehicle control, nor was there any significant interaction between time and treatment type (Fig. S1†). It should be noted that the statistical model used determines the change in weight over time by controlling for the impact of the animal's starting weight by using a random intercept structure.

The effect of the formulation on the different fat pads was also determined because of their important and often distinct roles in atherosclerosis.^18^ The formulation produced a significant increase in the weight of the renal fat pad (Fig. S2;†*p* = 0.027). In contrast, no significant changes were observed in the weight of subcutaneous fat, inguinal fat, gonadal fat, interscapular brown adipose tissue, thoracic perivascular adipose tissue (PVAT) or total fat (Fig. S2†).

Elevated levels of LDL/VLDL and total cholesterol are associated with an increased risk of CVD whereas high levels of HDL are considered as anti-atherogenic.^2–4^ The plasma levels of HDL showed a significant increase (*p* = 0.043) following feeding of a HFD with the formulation when compared to the vehicle control (Table 1). In addition, the formulation produced a trend towards reduction in plasma levels of total cholesterol (*p* = 0.076) and free cholesterol (*p* = 0.066) together with a non-significant decrease in LDL/VLDL (Table 1). No between group differences were observed for the total cholesterol : HDL and total cholesterol : LDL/VLDL ratios but a trend towards a decrease of the LDL/VLDL : HDL ratio (*p* = 0.077) was seen in mice receiving the formulation compared to the vehicle control (Table 1).

**Table 1:** Plasma lipids, oxidative markers and cytokines

	Control	Formulation	*p*-Value
**Lipids (mg dL** ^**−1**^ **)**			
TC	159 ± 10	136.7 ± 5.21	0.076
LDL/VLDL	53.59 ± 2.76	47.26 ± 3.32	0.174
HDL	40.12 ± 6.20	56.6 ± 3.45	0.043
TG	20.63 ± 1.16	40.38 ± 40.38	<0.001
FC	31.3 ± 5.66	15.92 ± 4.86	0.066
**Lipid ratios**			
TC : HDL	5.27 ± 1.83	2.46 ± 0.19	0.157
TC : LDL/VLDL	3.01 ± 0.27	2.94 ± 0.17	0.825
LDL/VLDL : HDL	1.61 ± 0.37	0.86 ± 0.09	0.077
TG : TC	0.15 ± 0.01	0.33 ± 0.04	<0.001
TG : HDL	0.74 ± 0.21	0.80 ± 0.09	0.790
TG : LDL/VLDL	0.44 ± 0.03	0.99 ± 0.14	0.004
**Oxidative markers**			
ROS (fold-change)	1.00 ± 0.05	1.16 ± 0.13	0.385
MDA (μM)	3.48 ± 0.78	7.20 ± 0.51	0.053
**Cytokines (pg ml** ^**−1**^ **)**			
CXCL1	159.60 ± 5.20	130.80 ± 13.80	0.106
IFN-γ	0.60 ± 0.20	0.80 ± 0.20	0.583
IL-1β	0.50 ± 0.10	0.40 ± 0.00	0.372
IL-5	2.00 ± 0.70	1.10 ± 0.10	0.243
IL-6	10.10 ± 3.20	5.40 ± 1.00	0.195
IL-10	9.20 ± 1.10	7.20 ± 1.00	0.243
TNF-α	7.50 ± 1.00	6.40 ± 0.30	0.110

The effect of the formulation on plasma TG levels was also determined as this can act as an independent risk factor for CVD.^10^ The formulation significantly increased plasma TG levels (*p* < 0.001) compared to the vehicle control (Table 1). The ratios of TG : total cholesterol, TG : HDL and TG : LDL/VLDL were also assessed. The TG : total cholesterol and TG : LDL/VLDL ratios were also significantly increased (*p* < 0.001 and *p* = 0.004 respectively) by the formulation with no significant changes for the TG : HDL ratio (Table 1).

*In vitro* experiments on human THP-1 macrophages carried out as our previous studies^15^ showed that the formulation (with or without PS) can increase TBHP-induced intracellular levels of ROS (Fig. S3†). In addition, such *in vitro* experiments with key active ingredients in the formulation showed that the TBHP-induced ROS production in THP-1 monocytes was significantly increased by EPA and DHA and significantly decreased by catechin and β-sitosterol (Fig. S4A†). In THP-1 macrophages, EPA, DHA and campesterol treatment significantly increased TBHP-induced ROS production whereas this was significantly attenuated by catechin (Fig. S4B†). In the light of these findings, the effect of the formulation on plasma ROS levels were determined *in vivo* and no significant differences between the groups were observed (Table 1). Increased lipid peroxidation is also pro-atherogenic and plasma malondialdehyde (MDA), a by-product of lipid peroxidation, is a biomarker of oxidative stress.^19^ The action of the formulation on plasma MDA levels was therefore also analyzed and showed an increase in mice receiving the formulation that almost reached significance (*p* = 0.053) (Table 1).

The plasma levels of several cytokines were determined because of their crucial roles in orchestrating the inflammatory response during atherosclerosis.^2,4,5,7^ The formulation produced a decrease in the plasma levels of several cytokines, including chemokine (C–X–C motif) ligand (CXCL)1, IL-1β, IL-5, IL-6, IL-10 and tumour necrosis factor (TNF)-α, though the changes were not significant (Table 1). No significant changes were seen in the ratio of pro-inflammatory : anti-inflammatory cytokine levels (data not shown).

### 3.2. The formulation produces a significant reduction in the frequency of macrophages and myeloid-derived suppressor cells (MDSC) within the bone marrow of mice fed a HFD

**Fig. 1:**
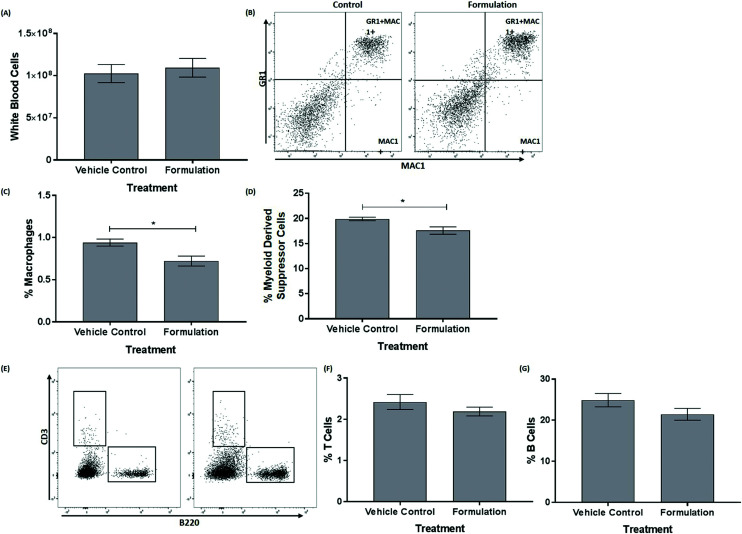
The formulation decreases the frequency of macrophages and MDSCs within the bone marrow. Cell populations were assessed in the bone marrow of mice after 21 days of a HFD and treatment with either a vehicle control or the formulation. (A) a bar graph showing numbers of white blood cells; (B) representative flow cytometry plot of macrophages and MDSCs; (C) frequency of macrophages in the bone marrow; (D) frequency of MDSCs in the bone marrow; (E) representative flow cytometry plot of T/B cells; (F) frequency of T cells in the bone marrow; and (G) frequency of B-cells in the bone marrow. The data in graphs are mean ± SEM from six control and seven formulation treated mice. Statistical analysis was performed using an unpaired Student's *t*-test (A, D, F and G) or Mann–Whitney *U* test (C) where * *p* ≤ 0.05.

**Fig. 2:**
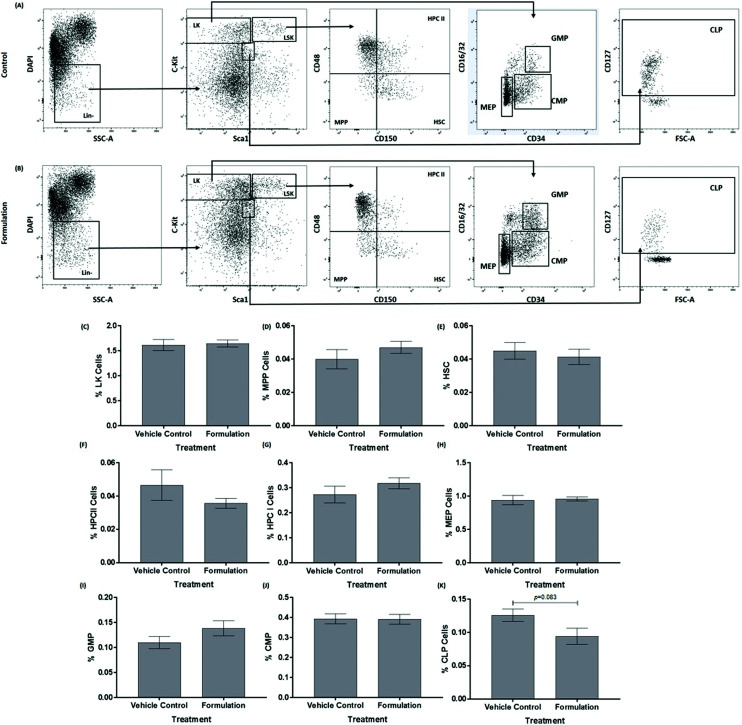
The effect of the formulation on hematopoietic stem and progenitor cells within the bone marrow. Cell populations were assessed in the bone marrow of mice after 21 days of a HFD and treatment with either a vehicle control (A) or the formulation (B). Representative flow cytometry plots of hematopoietic stem and progenitor cells in the bone marrow are presented (A and B). The bar graphs show the frequency of LK cells (C), MPP cells (D), HSC cells (E), HPCII cells (F), HPCI cells (G), MEP cells (H), GMP cells (I), CMP cells (J) and CLP cells (K) in the bone marrow. The data in graphs are mean ± SEM from six control and seven formulation treated mice (the various cell populations represent only a small proportion of starting cells). Statistical analysis was performed using an unpaired Student's *t*-test (C, D, E, G, H, I, J, K) or Mann–Whitney *U* test (F) and *p* values are stated where appropriate. Abbreviations: CLP, common lymphoid progenitor; CMP, common myeloid progenitor; GMP, granulocyte–macrophage progenitor; HPC, hematopoietic progenitor cell; HSC, hematopoietic stem cells; LK, Lin^−^ c-Kit^+^ cells; MEP, megakaryocyte-erythroid progenitor; MPP, multipotent progenitors.

The mature, stem and progenitor cell populations within the bone marrow of mice have been found to be altered following feeding of a HFD and changes in bone marrow cell populations have also been associated with the progression of atherosclerosis and metabolic syndrome.^20,21^ The effect of the formulation on the profile of these cells in the bone marrow was therefore determined. The data for the mature cells in the bone marrow are shown in Fig. 1. The formulation produced a significant decrease in the percentage of macrophages (*p* = 0.022, Fig. 1C) and MDSC (*p* = 0.023, Fig. 1D). No significant changes were seen for white blood cells (Fig. 1A), T-cells (Fig. 1F) and B-cells (Fig. 1G). For hematopoietic stem and progenitor cells, the formulation produced a trend towards decrease in the percentage of common lymphoid progenitor (CLP; *p* = 0.083, Fig. 2K) without significantly affecting Lin^−^ cKit^+^ (LK, Fig. 2C), multipotent progenitor cells (MPP, Fig. 2D), hematopoietic stem cells (HSC, Fig. 2E), hematopoietic progenitor cells (HPC) II (Fig. 2F), HPC I (Fig. 2G), megakaryocyte-erythroid progenitor (MEP, Fig. 2H) granulocyte-macrophage progenitor (GMP, Fig. 2I) and common myeloid progenitor (CMP, Fig. 2J).

### 3.3. The liver expression of several key genes implicated in atherosclerosis and metabolic syndrome was altered by the formulation

**Fig. 3:**
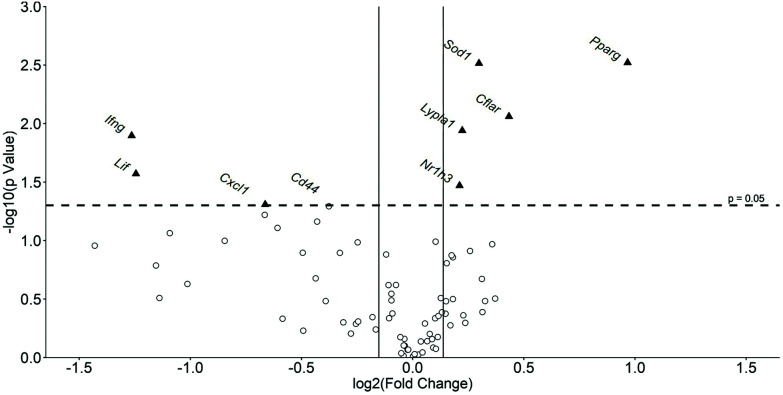
Volcano plot showing global gene expression changes in the liver of mice fed a HFD and treated with the formulation or vehicle control. Gene transcript levels of 84 genes were assessed in the livers of mice after 21 days of a HFD and treatment with either a vehicle control or the formulation. The data are presented as the mean formulation induced fold change when compared to the vehicle control treated mice. All genes present on the Qiagen RT^2^ profiler PCR Array were plotted. The log fold change following the treatment *vs*. the vehicle control cells is represented on the *x*-axis. The *y*-axis shows the –log 10 of the *p* value. A *p* value of 0.05 (dashed line) and a fold changes of ±10% (solid lines) are indicated. Genes whose expression was significantly altered by the formulation are indicated by a black triangle. Abbreviations: *Cd44*, CD44 antigen; *Cflar*, CASP8 and FADD-like apoptosis regulator; *Cxcl1*, Chemokine (C–X–C motif) ligand 1; *Ifng*, interferon-γ; *Lif*, leukemia inhibitory factor; *Lypla1*, lysophospholipase 1; *Nr1h3*, nuclear receptor subfamily 1; *Pparg*, peroxisome proliferator activator receptor-γ; *Sod1*, superoxide dismutase 1.

Liver gene expression has a major impact on changes in metabolism and inflammation seen in atherosclerosis and metabolic syndrome.^22,23^ The Mouse Atherosclerosis RT^2^ Profiler PCR Array was therefore used to investigate the effect of the formulation on the liver expression of 84 key genes implicated in the disease. Fig. 3 shows a volcano plot of changes in hepatic gene expression with Table 2 indicating percentage changes in the expression of all the genes analyzed. A total of 50 genes out of 84 were found to have their expression altered by at least 10% by the formulation. From these, the expression of 8 genes was significantly changed by the formulation with that for an additional gene, *Cd44*, almost reaching significance (*p* = 0.051) (highlighted in Fig. 3). The expression of five genes was significantly induced by the formulation: anti-atherogenic and anti-inflammatory transcription factors-*nuclear receptor subfamily 1 group H member 3* [*Nr1h3*; also called *liver-X-receptor* (*LXR*)-*α*]^24^ by 16% (*p* = 0.034) and *peroxisome proliferator-activated receptor (PPAR)-γ* (*Pparg*)^25^ by 95% (*p* = 0.003); the *Lypla1* gene that codes for acyl–protein thioesterase 1 (APT1),^26^ a lipase involved in lipid metabolism by 17% (*p* = 0.011); the *Sod1* gene that codes for superoxide dismutase (SOD)1, an enzyme capable of metabolising ROS^27^ by 23% (*p* = 0.003); and *CASP8 and FADD-like apoptosis regulator* [*Cflar*; also called *FLICE-like inhibitory protein* (*c-FLIP*)]^28^ implicated in the regulation of cell apoptosis by 35% (*p* = 0.009). In contrast, the expression of three genes was significantly attenuated by the formulation: *interferon-γ* (*Ifng*) (implicated in pro-inflammatory responses)^2,5,6^ by 58% (*p* = 0.013); *Cxcl1* (codes for pro-inflammatory chemokine)^5^ by 37% (*p* = 0.049); and *leukaemia inhibitory factor* (*Lif*) (implicated as anti-atherogenic cytokine)^5^ by 58% (*p* = 0.027). The formulation also produced a trend towards reduced expression of genes coding for *Cd44* implicated in cell–cell interactions, cell adhesion and migration^29^ by 23% (*p* = 0.051); *BCL2 related protein A1* (*Bcl2a1a*) by 53% (*p* = 0.086); *selectin P ligand* (*Selplg*) by 26% (*p* = 0.069); and *basic fibroblast growth factor* (*Fgf2*) by 34% (*p* = 0.078), (*Il1b* by 37% (*p* = 0.060) and *Tnf* by 44% (*p* = 0.101) (Table 2).

**Table 2:** The effect of the formulation on the expression of several key genes implicated in atherosclerosis

Gene	Control		Formulation		% Change	*p*-Value
	*N*	Mean ± SEM	*N*	Mean ± SEM		
*Abca1*	6	1 ± 0.04	6	1.13 ± 0.07	13 (↑)	0.139
*Ace*	5	1 ± 0.16	6	0.84 ± 0.18	16 (↓)	0.516
*Apoa1*	6	1 ± 0.04	6	0.94 ± 0.04	6 (↓)	0.285
*Apob*	6	1 ± 0.04	6	1.00 ± 0.02	0 (-)	0.977
*Apoe*	6	1 ± 0.07	6	1.05 ± 0.08	5 (↑)	0.629
*Bax*	6	1 ± 0.08	6	1.07 ± 0.04	7 (↑)	0.462
*Bcl2*	5	1 ± 0.16	6	0.74 ± 0.11	26 (↓)	0.210
*Bcl2a1a*	6	1 ± 0.24	6	0.47 ± 0.13	53 (↓)	0.086
*Bcl2l1*	6	1 ± 0.04	6	1.08 ± 0.09	8 (↑)	0.441
*Bid*	6	1 ± 0.05	6	1.04 ± 0.02	4 (↑)	0.512
*Birc3*	6	1 ± 0.03	6	0.94 ± 0.05	6 (↓)	0.325
*Ccl2*	6	1 ± 0.12	6	0.84 ± 0.18	16 (↓)	0.492
*Ccl5*	6	1 ± 0.21	6	0.76 ± 0.09	24 (↓)	0.329
*Ccr1*	6	1 ± 0.35	4	0.50 ± 0.17	50 (↓)	0.235
*Ccr2*	6	1 ± 0.21	6	1.29 ± 0.18	29 (↑)	0.313
*Cd44*	6	1 ± 0.09	6	0.77 ± 0.05	23 (↓)	0.051
*Cdh5*	6	1 ± 0.05	6	0.98 ± 0.07	2 (↓)	0.793
*Cflar*	6	1 ± 0.06	6	1.35 ± 0.09	35 (↑)	0.009
*Col3a1*	6	1 ± 0.16	6	1.25 ± 0.19	25 (↑)	0.329
*Csf2*	4	1 ± 0.32	2	0.77 ± 0.34	23 (↓)	NA
*Ctgf*	6	1 ± 0.48	6	0.45 ± 0.07	55 (↓)	0.310
*Cxcl1*	6	1 ± 0.14	5	0.63 ± 0.08	37 (↓)	0.049
*Eln*	6	1 ± 0.14	6	0.89 ± 0.12	11 (↓)	0.576
*Eng*	6	1 ± 0.06	6	0.98 ± 0.06	2 (↓)	0.858
*Fabp3*	5	1 ± 0.07	6	0.88 ± 0.13	12 (↓)	0.452
*Fas*	6	1 ± 0.06	6	1.11 ± 0.05	11 (↑)	0.156
*Fga*	6	1 ± 0.04	6	0.92 ± 0.03	8 (↓)	0.132
*Fgb*	6	1 ± 0.03	6	0.95 ± 0.03	5 (↓)	0.240
*Fgf2*	6	1 ± 0.15	5	0.66 ± 0.04	34 (↓)	0.078
*Fn1*	6	1 ± 0.05	6	0.93 ± 0.03	7 (↓)	0.240
*Hbegf*	6	1 ± 0.12	6	1.02 ± 0.22	2 (↑)	0.944
*Icam1*	6	1 ± 0.08	6	0.93 ± 0.05	7 (↓)	0.461
*Ifng*	5	1 ± 0.11	4	0.42 ± 0.13	58 (↓)	0.013
*Il1a*	6	1 ± 0.06	6	1.08 ± 0.17	8 (↑)	0.668
*Il1b*	6	1 ± 0.14	6	0.63 ± 0.10	37 (↓)	0.060
*Il1r1*	6	1 ± 0.09	6	1.18 ± 0.24	18 (↑)	0.506
*Il1r2*	6	1 ± 0.20	3	1.07 ± 0.20	7 (↑)	0.822
*Il2*	3	1 ± 0.59	3	0.97 ± 0.36	3 (↓)	0.970
*Il3*	4	1 ± 0.19	2	0.38 ± 0.05	62 (↓)	NA
*Il4*	4	1 ± 0.28	4	0.37 ± 0.03	63 (↓)	0.111
*Il5*	3	1 ± 0.44	3	0.23 ± 0.03	77 (↓)	0.225
*Itga2*	5	1 ± 0.13	4	0.97 ± 0.30	3 (↓)	0.919
*Itga5*	6	1 ± 0.09	6	1.28 ± 0.13	28 (↑)	0.108
*Itgax*	6	1 ± 0.23	6	0.81 ± 0.15	19 (↓)	0.500
*Itgb2*	6	1 ± 0.08	6	0.84 ± 0.03	16 (↓)	0.104
*Kdr*	6	1 ± 0.05	6	0.97 ± 0.03	3 (↓)	0.693
*Klf2*	0	NA	0	NA	NA	NA
*Lama1*	6	1 ± 0.10	6	1.05 ± 0.07	5 (↑)	0.725
*Ldlr*	6	1 ± 0.05	6	1.20 ± 0.10	20 (↑)	0.123
*Lif*	6	1 ± 0.19	5	0.42 ± 0.08	58 (↓)	0.027
*Lpl*	6	1 ± 0.11	6	1.12 ± 0.16	12 (↑)	0.531
*Lypla1*	6	1 ± 0.04	6	1.17 ± 0.02	17 (↑)	0.011
*Mmp1a*	6	1 ± 0.13	6	1.17 ± 0.16	17 (↑)	0.436
*Mmp3*	5	1 ± 0.35	3	0.67 ± 0.24	33 (↓)	0.466
*Msr1*	6	1 ± 0.04	6	0.96 ± 0.08	4 (↓)	0.670
*Nfkb1*	6	1 ± 0.03	6	1.07 ± 0.03	7 (↑)	0.102
*Npy*	1	1 ±NA	0	NA	NA	NA
*Nr1h3*	6	1 ± 0.05	6	1.16 ± 0.03	16 (↑)	0.034
*Pdgfa*	6	1 ± 0.10	6	1.06 ± 0.12	6 (↑)	0.696
*Pdgfb*	6	1 ± 0.11	6	0.80 ± 0.04	20 (↓)	0.128
*Pdgfrb*	6	1 ± 0.07	5	1.03 ± 0.02	3 (↑)	0.729
*Plin2*	6	1 ± 0.09	6	1.11 ± 0.06	11 (↑)	0.330
*Ppara*	6	1 ± 0.08	6	1.13 ± 0.09	13 (↑)	0.317
*Ppard*	6	1 ± 0.07	6	0.99 ± 0.02	1 (↓)	0.857
*Pparg*	6	1 ± 0.10	6	1.95 ± 0.20	95 (↑)	0.003
*Ptgs1*	6	1 ± 0.04	6	1.01 ± 0.06	1 (↑)	0.935
*Rxra*	6	1 ± 0.04	6	1.13 ± 0.06	13 (↑)	0.134
*Sele*	5	1 ± 0.31	6	1.08 ± 0.19	8 (↑)	0.842
*Sell*	5	1 ± 0.45	6	0.71 ± 0.24	29 (↓)	0.590
*Selp*	6	1 ± 0.05	6	0.94 ± 0.06	6 (↓)	0.420
*Selpg*	6	1 ± 0.11	6	0.74 ± 0.04	26 (↓)	0.069
*Serpinb2*	4	1 ± 0.29	4	0.45 ± 0.16	55 (↓)	0.164
*Serpine1*	6	1 ± 0.09	6	1.24 ± 0.15	24 (↑)	0.213
*Sod1*	6	1 ± 0.05	6	1.23 ± 0.03	23 (↑)	0.003
*Spp1*	6	1 ± 0.10	6	1.11 ± 0.09	11 (↑)	0.422
*Tgfb1*	6	1 ± 0.05	6	1.09 ± 0.07	9 (↑)	0.310
*Tgfb2*	5	1 ± 0.30	6	0.82 ± 0.15	18 (↓)	0.625
*Thbs4*	5	1 ± 0.52	0	NA	NA	NA
*Tnc*	6	1 ± 0.21	6	1.24 ± 0.19	24 (↑)	0.409
*Tnf*	6	1 ± 0.20	5	0.56 ± 0.14	44 (↓)	0.101
*Tnfaip3*	6	1 ± 0.12	6	1.03 ± 0.22	3 (↑)	0.905
*Vcam1*	6	1 ± 0.07	6	0.97 ± 0.08	3 (↓)	0.788
*VeffA*	6	1 ± 0.09	6	1.10 ± 0.07	10 (↑)	0.410
*Vwf*	6	1 ± 0.16	6	0.71 ± 0.05	29 (↓)	0.127
*Actb*	6	1 ± 0.02	6	1.08 ± 0.06	8 (↑)	NA
*B2m*	6	1 ± 0.05	6	0.95 ± 0.01	5 (↓)	NA
*Gapdh*	6	1 ± 0.04	6	0.92 ± 0.03	8 (↓)	NA
*Gusb*	6	1 ± 0.04	6	0.98 ± 0.05	2 (↓)	NA
*Hsp90ab1*	6	1 ± 0.03	6	1.09 ± 0.04	9 (↑)	NA

## 4. Discussion

The anti-atherogenic effects of fish oils, cocoa extracts and PS are well documented^3,4^ but no study has examined the effect of a combination of these active ingredients in their natural or most available format *in vivo*. In this study in C57BL6/J mice fed a HFD for 3 weeks, supplementation with the emulsified formulation increased plasma HDL levels and improved the LDL/VLDL : HDL ratio (Table 1). Anti-atherogenic effects were also observed on cell populations within the bone marrow (*e.g.* reduced levels of macrophages; Fig. 1) together with the beneficial modulation of expression of both pro- and anti-atherogenic genes in the liver (*e.g.* increased expression of anti-atherogenic genes *Nr1h3*, *Pparg* and *Sod1* together with decreased expression of pro-atherogenic genes such as *Ifng* and *Cxcl1*) (Fig. 3 and Table 2). The study extends our previous *in vitro* research on the protective actions of the formulation on several monocyte/macrophage processes associated with atherosclerosis *in vitro*^15^ to an *in vivo* context (*e.g.* beneficial effects on risk factors associated with atherosclerosis and metabolic syndrome such as plasma lipoprotein profile, systemic inflammation, hepatic lipid alterations and gene expression) and supports further studies on mouse model systems for these diseases and clinical trials.

The formulation had no impact on the weight gain of mice during 3 weeks feeding of HFD (Fig. S1†). Previous studies on EPA, DHA or fish oils showed no short-term differences (3–4 weeks) but a reduction in body weight following 10–12 weeks of feeding,^30,31^ suggesting that longer feeding periods may be required for beneficial weight changes. Components of the formulation have been shown to impact upon various fat stores within the body though the results are not always consistent.^31–34^ For example, previous studies have found that both omega-3 PUFAs and flavanol supplementation resulted in reduced white fat accumulation *in vivo*^31,32^ whereas others have shown that both flavanols and PS had no effect on the size of the white fat deposits.^33,34^ The only significant change in fat stores produced by the formulation was an increase in renal fat weight with a non-significant reduction in interscapular brown fat and increase in thoracic PVAT (Fig. S2†). The expansion of PVAT is considered anti-atherogenic as it is known to contribute to fatty acid clearance *via* thermogenesis and reducing inflammation, and loss of PVAT in mouse model systems has been associated with increased atherosclerosis.^18^ On the other hand, reduced weight of interscapular brown fat pads has been shown to occur during brown fat activation and contributes to the attenuation of atherosclerosis development in mouse model systems.^35^ The short term activation of brown fat is associated with increases in TG levels,^36^ expression of *Nr1h3* and *Pparg*,^37,38^ HDL cholesterol levels^36^ and lipid perxodation.^39^ These changes are consistent with the findings of our study and thus suggest a potential mechanism that needs to be investigated in future studies with model systems involving HFD feeding for longer duration (*e.g.* 12 weeks).

Increased plasma HDL levels produced by the formulation is a key anti-atherogenic event because of its important role in promoting reverse cholesterol transport and acting in an anti-inflammatory manner.^2–4^ Indeed, our previous *in vitro* study showed that this formulation stimulated macrophage cholesterol efflux *in vitro*.^15^ Future studies should investigate the effect of the formulation on the expression of lipoprotein associated enzymes such as phospholipase A_2_ (HDL-LpPLA_2_) and paraoxonase-1 (PON1). Studies in mouse models of atherosclerosis have also shown that EPA dietary supplementation for 28 days increased plasma HDL levels.^40^ In addition, increased HDL levels were observed in rats and mice treated with a flavonol-rich green tea extract or with PS.^41,42^ In contrast to HDL, the formulation produced non-significant reduction of plasma levels of LDL/VLDL (Table 1). This finding contradicts previous studies that have shown that omega-3 PUFAs, flavanols and PS are all capable of significantly reducing plasma LDL levels.^4,42^ However, the formulation did produce a trend towards reduction of LDL/VLDL : HDL ratio (Table 1) and there is growing evidence that assessing LDL or HDL cholesterol levels in isolation is not always associated with cardiovascular health and the LDL : HDL ratio is a better clinical risk marker of atherosclerosis and sudden cardiac death.^43,44^ The formulation caused an increase in plasma TG levels (Table 1), which is surprising as previous studies have shown that omega-3 PUFAs and PS reduced circulating TG levels^3,4,10,45^ though flavanols have failed to significantly alter TG levels in both mice and humans.^4,5,46^ However, TGs are not thought to play a direct role in atherosclerosis disease development^47^ and, as discussed above, increased plasma TG levels may be indicative of brown fat activation that is known to protect against disease development. The discrepancies also point that combinations of nutritionally active ingredients in their natural context have different actions compared to individual components, thereby indicating the need for more detailed studies on combination products. Several examples of such differences between combination products and individual components exist; for example, extra virgin olive oil reduces atherosclerosis in apolipoprotein E deficient mouse model system whereas its major polyphenol, hydroxytyrosol, increases plaque burden in the same model.^48,49^ In addition, differential effects of EPA and DHA present in fish oils have been documented.^50^

Several recent *in vivo* studies have demonstrated that omega-3 PUFAs, flavanols and PS dietary supplementation are capable of attenuating oxidative stress.^3,4,51,52^ Our *in vitro* studies showed that the formulation increased macrophage ROS production (Fig. S3†) though this was not found to be the case *in vivo* (Table 1). However, the formulation did increase lipid peroxidation, a consequence of increased ROS concentration, as measured by plasma MDA levels (Table 1). This result differs from other studies which have shown that omega-3 PUFAs, PS and flavanols attenuate lipid peroxidation^51–53^ but further points to potential brown fat activation^39^ that, as detailed above, needs to be investigated as part of future research as this is associated with increased *Sod1* expression (Table 2) and lipid peroxidation (Table 1) as seen in this study.

The inflammatory response in atherosclerosis is orchestrated by a multitude of cytokines.^5–8^ Dietary supplementation with omega-3 PUFAs, flavanols and PS has been found to reduce the circulating levels of CXCL1 and TNF-α in mice.^4,54,55^ In addition, omega-3 PUFAs reduced the levels of IL-5 and IL-10 in mice whereas flavanol treatment failed to alter IL-10 levels and the effects of PS were more complex with IL-5 levels being attenuated and IL-10 levels unaffected.^3,4,56–59^ In this study, no significant changes in the plasma levels of these or other cytokines were observed in response to the formulation and with the exception of IFN-γ, the plasma levels of all the cytokines analyzed were reduced by supplementation with the formulation (Table 1). This suggests a dampening of immune activation and supports the observed reductions in atherogenic immune cell populations in the bone marrow (Fig. 1). Mature, stem and progenitor cell populations within the bone marrow of mice have been found to be altered following feeding of a HFD and during the development of atherosclerosis.^20,21^ In this study, the formulation produced a significant reduction in the percentage of macrophages and MDSCs within the bone marrow (Fig. 1). The pro-atherogenic roles of macrophages in atherosclerosis is well established.^5^ The role of MDSC in inflammation and atherosclerosis is less well defined^60^ and the reduction on their population may be linked to changes in IL-6 and IL-10 (Table 1) that are known to be involved in the differentiation of MDSCs.^60^

The liver also has a major impact upon inflammation, metabolism and atherosclerosis^22,23^ and the formulation were found to modulate the expression of a number of hepatic genes that further supports its anti-atherogenic potential. The expression of five genes (*Cflar*, *Lypla1*, *Nr1h3*, *Ppard* and *Sod1*) was significantly induced by the formulation. CFLAR can prevent cell apoptosis by blocking caspase 8 activation and stimulating nuclear factor kappa B (NFκB) and extracellular signal-regulated kinase (ERK) signaling^28^ and its expression is elevated in healthy hearts and reduced following heart failure.^61^ In addition, oxLDL attenuated *Cflar* expression in vascular endothelial cells leads to apoptosis.^61^ Previous research has shown that omega-3 PUFAs promote *Cflar* expression whereas the effects of flavanols and PS are not known.^62^ The transcription factors NR1H3 (LXR α) and PPAR-γ have been demonstrated to have anti-inflammatory and anti-atherogenic actions.^24,25^ Both NR1H3 and PPAR-γ protect against cholesterol accumulation within cells by enhancing the expression of genes involved in cholesterol efflux and transportation, and attenuating the expression of pro-inflammatory genes, including *Tnf-α* and *Il-1β*,^24,25^ which is consistent with the apparent reduction in the plasma levels of these cytokines (Table 1). NR1H3 activation is also associated with reduced dietary cholesterol absorption from the intestine and elevation of HDL cholesterol levels,^24^ and may therefore contribute to our observed increase in plasma HDL (Table 1). In addition to regulating cholesterol homeostasis, NR1H3 induces the hepatic transcription of several lipogenic enzymes.^24^ PPAR-γ is also well known target for antidiabetic drugs such as thiazolidiniones.^25^ Thus, future studies of HFD feeding of longer duration (*e.g.* 12 weeks) should investigate the effect of the formulation on markers of hepatic steatosis and improvement in glucose homeostasis.

SOD1 is considered anti-atherogenic because of its ability to neutralize free superoxide radicals^27^ and increased hepatic *Sod1* expression in response to the formulation may explain in part why ROS generation was not significantly increased *in vivo* (Table 1) despite increases seen *in vitro* (Fig. S3 and S4†) though potential antagonism between different active ingredients could have contributed. The enzyme APT1 is encoded by the gene *Lypla1* which was also found to have its expression induced by the formulation (Table 2). Although the function of APT1 during atherosclerosis disease development remains unclear, it is involved in lipid metabolism (depalmitoylation).^26^

From the down regulated genes, the chemokine CXCL1 plays an important role in the recruitment of monocytes to the site of oxLDL accumulation in atherosclerosis and its circulating levels are increased in patients with coronary artery disease.^5^ The reduced hepatic expression of this gene in our study together with the apparent reduced plasma levels (Table 1) is consistent with the decrease in monocyte migration by the formulation observed in our previous *in vitro* study.^15^ In addition, CD44, ITGB2 and SELPLG are all known to play a role in cell recruitment during atherosclerosis.^29,63^ IFN-γ is an important pro-atherogenic cytokine and reduced expression of the gene has been observed during *in vivo* assessment of the effects of individual ingredients present in the formulation^40,64,65^ although reductions in the plasma levels of this cytokine were not observed in our study. Finally, not all gene expression changes were anti-atherogenic; for example, consistent with previous studies on flavanols,^66^ the expression of *Lif* was also reduced by the formulation (Table 2). LIF is able to attenuate endothelial cell proliferation, induce vasorelaxation within arteries by increasing nitric oxide production and inhibit vascular smooth muscle cell proliferation.^5^

## 5. Conclusions

This is the first *in vivo* study to assess the effect of a unique nutritional combination containing omega-3 PUFAs, flavanols and PS at a human physiological equivalent dose in mice receiving a HFD. The formulation has shown promising potential in reducing risk factors associated with atherosclerosis and metabolic syndrome in the light of its ability to increase plasma HDL levels, promote an anti-atherogenic cell profile in the bone marrow and beneficially impact both hepatic gene expression and lipid alterations. Furthermore, there is an indication that the formulation is potentially capable of enhancing brown fat activation as suggested by changes in gene expression and several other plasma parameters that needs to be investigated as part of future studies.

The number of animals used and the duration of HFD feeding in the current study is similar to our previous published research that demonstrated many beneficial anti-atherogenic changes in risk factors,^16^ some of which were subsequently translated into humans.^67^ Limitations of the current study include the number of animals used, which may have contributed in part for some of the trends not reaching statistical significance, the short duration of HFD feeding and the use of only male mice. Future studies should be carried out on a larger number of both male and female mice (to delineate any gender-specific differences), using both short-term HFD feeding as in this study and for a longer period (*e.g.* 12 weeks) and compare the action of the formulation with individual components. The studies should also be extended to mouse models of atherosclerosis where the efficacy of the formulation and individual components to attenuate the development of the disease and cause regression of existing plaques can be investigated together with impact on hepatic steatosis, glucose homeostasis and brown fat activation. Finally, the efficacy of the formulation to reduce CVD burden and associated risk factors should be analyzed in clinical trials.

## Abbreviations

APT1: Acyl-protein thioesterase 1
ANOVA: One-way analysis of variance
APC: Allophycocyanin
Apo: Apolipoprotein
B2M: β-2-Microglobulin
BCL2A1A: BCL2 related protein A1
CFLAR: CASP8 and FADD-like apoptosis regulator
c-FLIP: FLICE-like inhibitory protein
CMP: Common myeloid progenitor
CVD: Cardiovascular disease
CLP: Common lymphoid progenitor
CXCL: Chemokine (C–X–C motif) ligand
Cy7: Cyanine7
DHA: Docosahexaenoic acid
ECM: Extracellular matrix
EPA: Eicosapentaenoic acid
FGF2: Basic fibroblast growth factor
GMP: Granulocyte-macrophage progenitor
GUSB: β-Glucuronidase
HDL: High density-lipoprotein
HFD: High fat diet
HI-FCS: Heat-inactivated fetal calf serum
HPC: Hematopoietic progenitor cell
HSC: Hematopoietic stem cell
HSP90AB1: Heat shock protein HSP 90-β
IL: Interleukin
LIF: Leukaemia inhibitory factor
LDL: Low-density lipoprotein
LK: Lin^−^ Sca-1^−^ c-Kit^+^
LSK: Lin^−^ Sca-1^+^ c-Kit^+^
MDSC: Myeloid-derived suppressor cells
MEP: Megakaryocyte-erythroid progenitor
MDA: Malondialdehyde
MI: Mycocardial infarction
MPP: Multipotent progenitors
NR1H3: Nuclear receptor subfamily 1 group H member 3
ox: Oxidized
PE: Phycoerythrin
PPAR: Peroxisome proliferator-activated receptor
PS: Phytosterols
PUFA: Polyunsaturated fatty acids
PVAT: Perivascular adipose tissue
REDUCE-IT: Reduction of cardiovascular events with icosapent ethyl–intervention trial
ROS: Reactive oxygen species
SELPLG: Selectin P ligand
SLAM: Signaling lymphocytic activation molecule
SOD: Superoxide dismutase
TBHP: *tert*-Butyl hydroperoxide
TG: Triglycerides
TNF: Tumor necrosis factor
VLDL: Very low-density lipoprotein

## Author contributions

JWEM, JOW, TRH, JBMG, SFP, NPR, DRM and DPR were responsible for the design of the experiments. Experiments were performed by JWEM, JOW, WA, TRH, and JBMG. Data analysis was carried out by JWEM, JOW, JBMG, VOM, YHC and AA. JWEM, JOW and YHC prepared the figures and JWEM and DPR wrote the manuscript. All the authors contributed to the review of the manuscript.

## Conflicts of interest

This study was supported by Cultech Ltd, Port Talbot, UK. DRM and SFP are employees of Cultech Ltd. JWEM PhD was funded by a joint studentship from the School of Biosciences, Cardiff University and Cultech Ltd.

## Supplementary Material

FO-012-D0FO02867C-s001
