# Supplementary material for: Protective effects of a unique combination of nutritionally active ingredients on risk factors and gene expression associated with atherosclerosis in C57BL/6J mice fed a high fat diet
Source: Food Funct. 2021 Mar 9;12(8):3657–71. doi: 10.1039/d0fo02867c (PMC8359826; doi:10.1039/d0fo02867c)
Supplement: FO-012-D0FO02867C-s001 [file FO-012-D0FO02867C-s001.pdf]

Electronic Supplementary Material (ESI) for Food & Function.
This journal is © The Royal Society of Chemistry 2021

Supplementary Data:

Protective effects of a unique combination of nutritionally active ingredients on risk factors and gene expression associated with atherosclerosis in C57BL/6J mice fed a high fat diet

Joe W.E. Moss[1], Jessica O. Williams[1], Wijdan Al-Ahmadi[1], Victoria O'Morain[1], Yee-Hung Chan[1], Timothy R. Hughes[2], Juan B. Menendez-Gonzalez[3], Alhomidi Almotiri[3], Sue F. Plummer[4], Neil P. Rodrigues[3], Daryn R. Michael[4] and Dipak P. Ramji[1*].

[1]Cardiff School of Biosciences, Cardiff University, Sir Martin Evans Building, Museum Avenue, Cardiff CF10 3AX, UK.

[2]Systems Immunity Research Institute, School of Medicine, Cardiff University, Cardiff CF14 4XN, UK.

[3]European Cancer Stem Cell Research Institute, Cardiff School of Biosciences, Cardiff University, Hadyn Ellis Building, Maindy Road, Cardiff CF24 4HQ, UK.

[4]Cultech Limited, Unit 2 Christchurch Road, Baglan Industrial Park, Port Talbot, SA12 7BZ, UK.

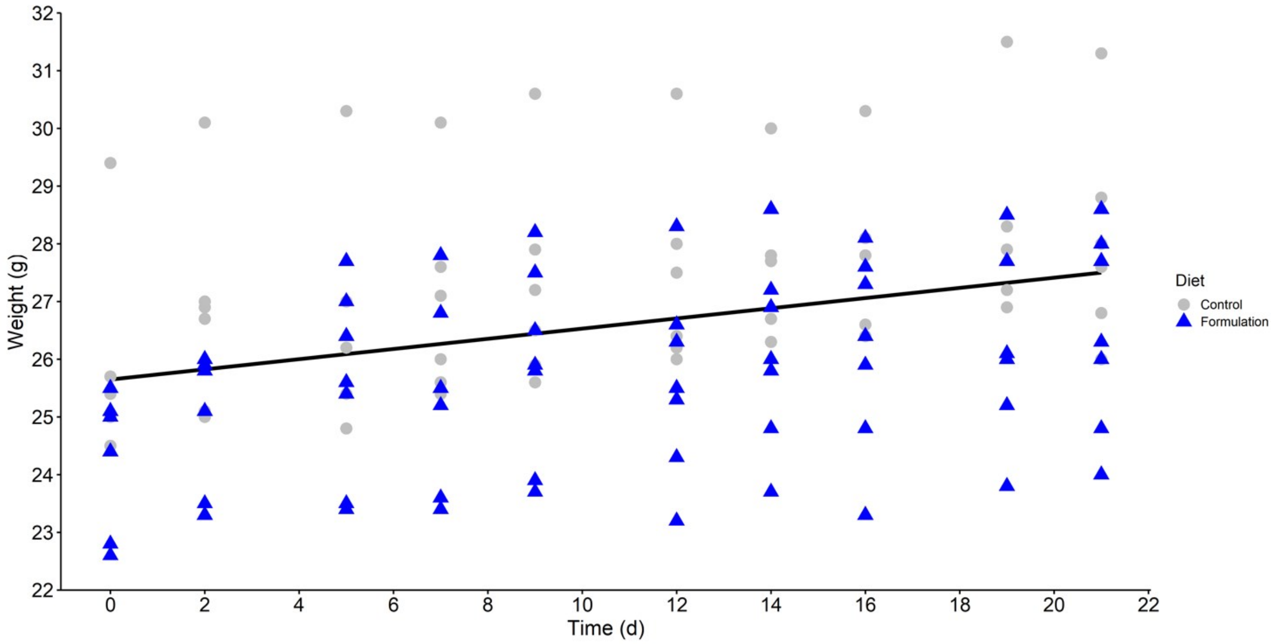

Figure S1. The rate of weight gain in C57BL/6J mice fed a HFD was not altered by the formulation

The weight of the mice was measured approximately every 2 days for the entire duration of the study (21 days). Mice were fed a HFD which was either supplemented with the vehicle control (circle) or a 1x human equivalent physiological dose of the formulation (triangle). The prediction of the average rate of weight gain, as calculated by a generalized linear mixed model with a Gamma error distribution and identity link function, is displayed from 13 mice (control n=6; formulation n=7). The raw data from each mouse are also presented to provide a visual representation of the spread within the data. The initial statistical model showed that there was no significant effect for the formulation treatment or the interaction between time and treatment type. Therefore, they were removed from the final statistical model during model refinement.

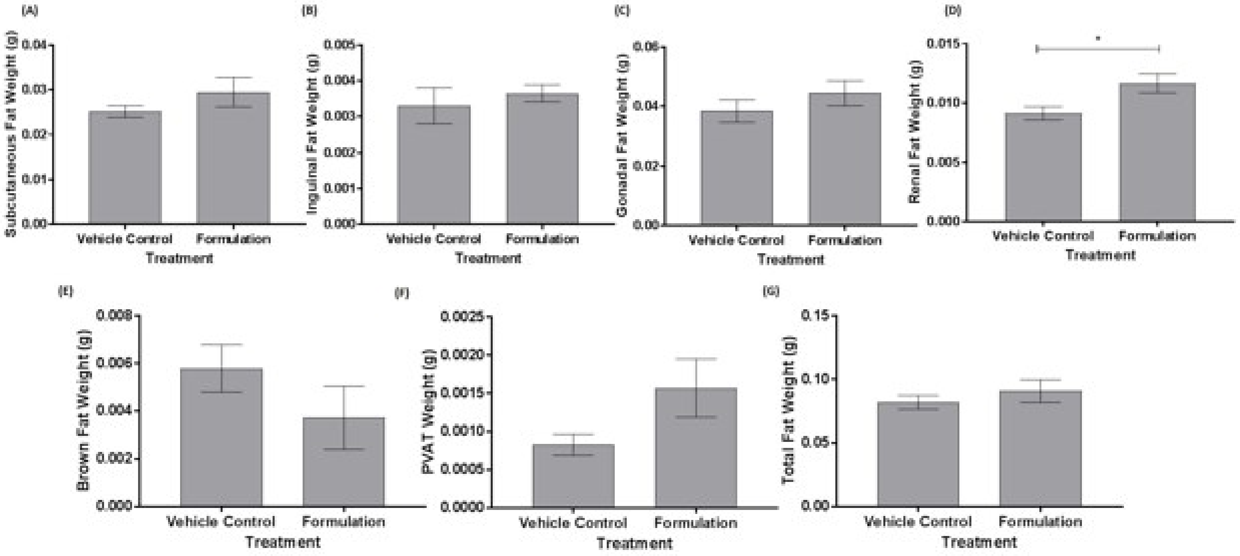

Figure S2. The formulation increases the weight of renal fat pad in C57BL/6J mice fed a HFD

The subcutaneous (A), inguinal (B), gonadal (C), renal (D), interscapular brown (E), thoracic perivascular adipose tissue (PVAT) (F) and total (G) fats from C57BL/6J mice were weighed after 21 days of a HFD and treatment with either a vehicle control or a 1x human equivalent physiological dose of the formulation, and normalized to the total body weight of individual mice. The data are mean ± SEM from 13 mice (control n=6; formulation n=7). Statistical analysis was performed using an unpaired Student's t-test (equal variance) on either untransformed data (C, D, E and F) or log-transformed data (A, B and G) where * $p \leq 0.05$.

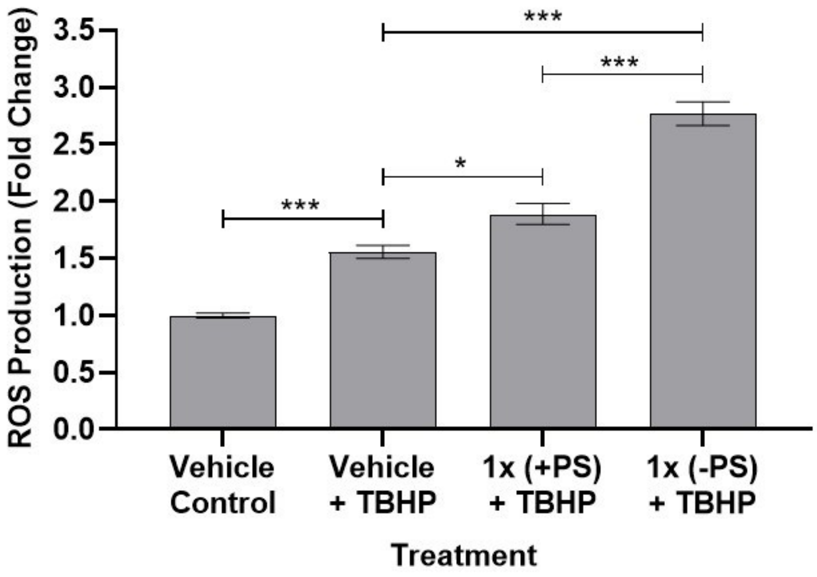

Figure S3. The formulation induces ROS production in human macrophages

ROS production was assessed in phorbol-12-myristate-13-acetate differentiated THP-1 macrophages that were either treated with vehicle (vehicle control) or tert-Butyl hydroperoxide (TBHP; 50 μM) or TBHP (50 μM) in the presence of the formulation containing phytosterols (+PS) or TBHP (50 μM) in the presence of the formulation lacking phytosterols (-PS) for 3 h. ROS production was measured in a fluorescence microplate reader, with excitation at 485 nm and emission detected at 535 nm. The value obtained with the vehicle control was arbitrary assigned as 1. The data are mean ± SEM from three independent experiments. Statistical analysis was performed using a One-way ANOVA with Tukey's post-hoc analysis where * $p \leq 0.05$ and *** $p \leq 0.001$.

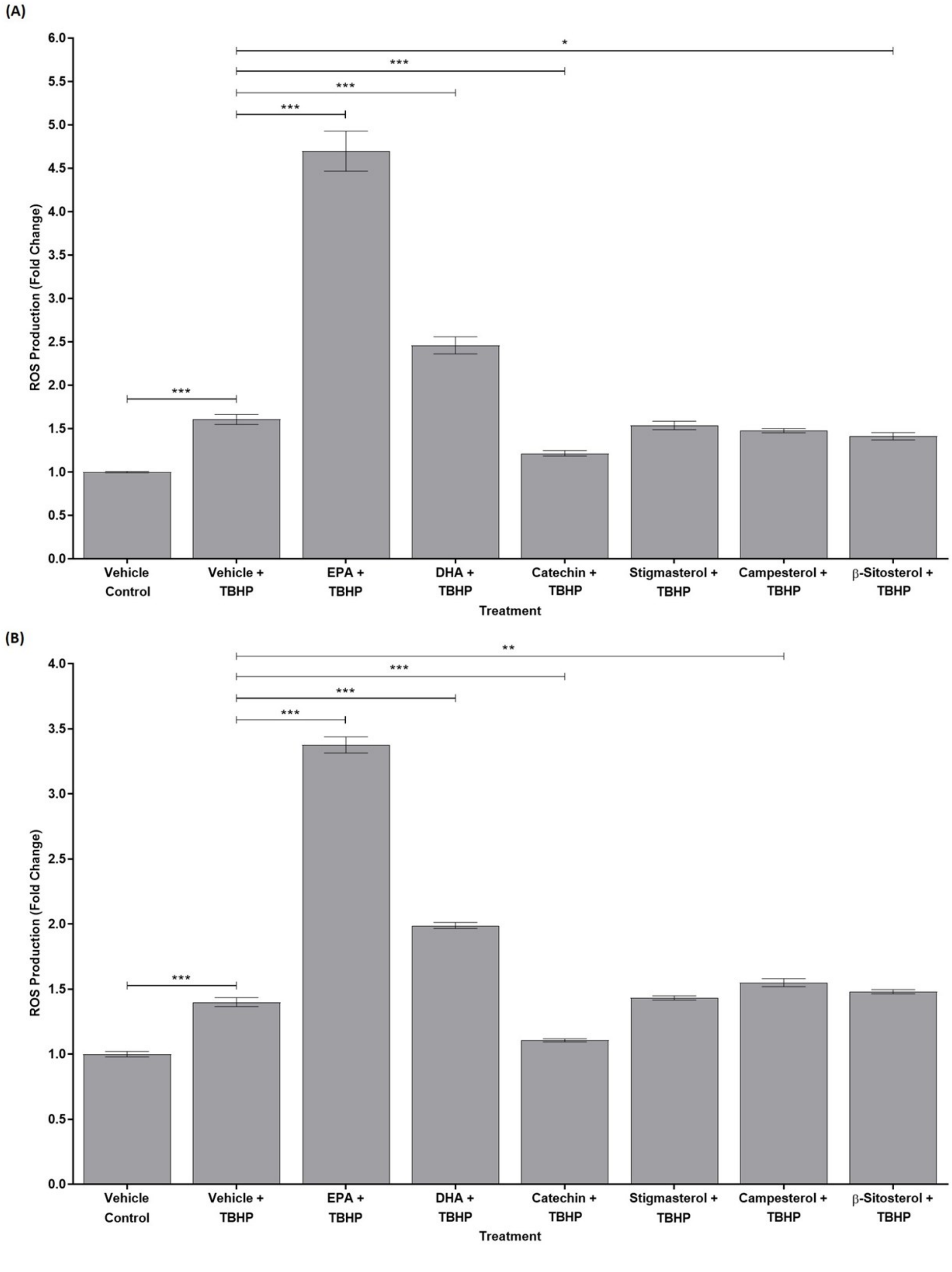

Fig. S4. Key ingredients in the formulation can modify ROS production in human monocytes and macrophages.

ROS production was assessed in THP-1 monocytes (A) and THP-1 macrophages (B) that were either treated with vehicle (vehicle control) or TBHP; 50 μM) or TBHP (50 μM) in the presence 30 μg/ml EPA, 19.7 μg/ml DHA, 1.5 μg/ml catechin, 10 μg/ml stigmasterol, 13.9 μg/ml campesterol or 27.2 μg/ml β-sitosterol. ROS production was measured in a fluorescence microplate reader, with excitation at 485 nm and emission detected at 535 nm. The value obtained with the vehicle control was arbitrary assigned as 1. The data are mean ± SEM from three independent experiments. Statistical analysis was performed on the log-transformed data using a One-way ANOVA with Dunnett post-hoc analysis where * $p \leq 0.05$, ** $p \leq 0.01$ and *** $p \leq 0.001$.

Table S1: Details of reagents used for flow cytometry

Reagent	Fluorochrome where applicable	Supplier	Clone	Catalogue number
CD117 (c-Kit)	APC	BioLegend	2B8	105811
Ly-6A/E (Sca-1)	PE	BioLegend	D7	108107
CD48	FITC	BioLegend	HM48-1	103403
CD150	PE/Cy7	BioLegend	TC15-12F12.2	115913
Ly-6A/E (Sca-1)	APC/Cy7	BioLegend	D7	108125
CD34	FITC	BD Biosciences	RAM34	560238
CD16/32	PE/Cy7	BioLegend	93	101317
CD127 (IL-7Rα)	PE	BioLegend	SB/199	121111
Ly-6G/Ly-6C (Gr-1)	PE/Cy7	BioLegend	RB6-8C5	108415
CD11b (Mac-1)	PE	BioLegend	M1/70	101207
CD45R/B220	APC	BioLegend	RA3-6B2	103212
CD3	FITC	BioLegend	17A2	100203
TER-119	APC/Cy7	BioLegend	TER-119	116223
Biotin CD3		BioLegend	17A2	100244
Biotin CD4		BioLegend	GK1.5	100404
Biotin CD8a		BioLegend	53-6.7	100703
Biotin Ly-6G/Ly-6C (Gr-1)		BioLegend	RB6-8C5	108404
Biotin CD11b		BioLegend	M1/70	101204
Biotin CD45R/B220		BioLegend	RA3-6B2	103204
Biotin TER-119		BioLegend	TER-119	116204
TruStain fcX™ (CD16/32)		BioLegend	93	101319
